# Radiosurgery or hypofractionated stereotactic radiotherapy for brain metastases from radioresistant primaries (melanoma and renal cancer)

**DOI:** 10.1186/s13014-018-1083-1

**Published:** 2018-07-28

**Authors:** Paul Lesueur, Justine Lequesne, Victor Barraux, William Kao, Julien Geffrelot, Jean-Michel Grellard, Jean-Louis Habrand, Evelyne Emery, Brigitte Marie, Juliette Thariat, Dinu Stefan

**Affiliations:** 1 Radiotherapy department, Centre François Baclesse, Caen, France; 2 Laboratoire d’accueil et de recherche avec les ions accélérés, CEA-CIMAP, Caen, France; 3 Clinical research department, Centre François Baclesse, Caen, France; 4 Medical physics department, Centre François Baclesse, Caen, France; 5 Neurosurgery department, CHU Côte de Nacre, Caen, France; 6 Imaging department, Centre François Baclesse, Caen, France; 7 Medical university of Caen, Caen, France

**Keywords:** Stereotactic radiotherapy, Brain metastases, Radioresistant, Melanoma, Fractionation, Renal Cancer

## Abstract

**Background:**

Until 50% of patients with renal cancer or melanoma, develop brain metastases during the course of their disease. Stereotactic radiotherapy has become a standard of care for patients with a limited number of brain metastases. Given the radioresistant nature of melanoma and renal cancer, optimization of the fractionation of stereotactic radiotherapy is needed. The purpose of this retrospective study was to elucidate if hypofractionated stereotactic radiotherapy (HFSRT) impacts local control of brain metastases from radioresistant tumors such as melanoma and renal cancer, in comparison with radiosurgery (SRS).

**Methods:**

Between 2012 and 2016, 193 metastases, smaller than 3 cm, from patients suffering from radioresistant primaries (melanoma and renal cancer) were treated with HFSRT or SRS. The primary outcome was local progression free survival (LPFS) at 6, 12 and 18 months. Overall survival (OS) and cerebral progression free survival (CPFS) were secondary outcomes, and were evaluated per patient. Objective response rate and radionecrosis incidence were also reported. The statistical analysis included a supplementary propensity score analysis to deal with bias induced by non-randomized data.

**Results:**

After a median follow-up of 7.4 months, LPFS rates at 6, 12 and 18 months for the whole population were 83, 74 and 70%, respectively. With respect to fractionation, LPFS rates at 6, 12 and 18 months were 89, 79 and 73% for the SRS group and 80, 72 and 68% for the HFSRT group. The fractionation schedule was not statistically associated with LPFS (HR = 1.39, CI95% [0.65–2.96], *p* = 0.38). Time from planning MRI to first irradiation session longer than 14 days was associated with a poorer local control rate. Over this time, LPFS at 12 months was reduced from 86 to 70% (*p* = 0.009). Radionecrosis occurred in 7.1% for HFSRT treated metastases to 9.6% to SRS treated metastases, without any difference according to fractionation (*p* = 0.55). The median OS was 9.6 months. Six, 12 and 18 months CPFS rates were 54, 24 and 17%, respectively.

**Conclusion:**

Fractionation does not decrease LPFS. Even for small radioresistant brain metastases (< 3 cm), HFSRT, with 3 or 6 fractions, leads to an excellent local control rate of 72% at 1 year with a rate of 7.1% of radionecrosis. HFSRT is a safe and efficient alternative treatment to SRS.

**Electronic supplementary material:**

The online version of this article (10.1186/s13014-018-1083-1) contains supplementary material, which is available to authorized users.

## Background

About 10 and 50% of patients with renal cancer [1] or melanoma [2], develop brain metastases during the course of their disease respectively. Multimodality treatment including neurosurgery, radiotherapy, systemic treatments, and best supportive care with respect to disease burden, patient preference and performance status is usual. Stereotactic radiotherapy has become a standard of care for patients with a limited number of brain metastases [3, 4]. This is so regardless a histology-based approach despite different spread patterns and different response to the various treatment modalities. On the basis of such results, in our institution, we treat patients having up to 10 brain metastases from melanoma or renal cancer with stereotactic radiotherapy. Given the radioresistant nature of melanoma and renal cancer, optimization of the fractionation of stereotactic radiotherapy is needed. Following the linear quadratic model [5], and considering an α/β ratio about 2.5 [6, 7], then biological effective dose is much higher with SRS (>18Gy) than with HFSRT. Thus, it was expected that brain metastases of radioresistant primary tumors treated with HSFRT would show poorer local control than these treated with SRS. Indeed, a retrospective study with a small sample concluded that fractionated stereotactic radiotherapy was less effective in radioresistant tumors and recommended that radioresistant tumors should be treated in a single fraction if possible [8, 9]. The linear quadratic model has been suggested to be incorrect for large dose fractions, because it does not take into account additional biological effects resulting from high dose on endothelial cells and tumor immunity. Thus, local control could be not strictly correlated to calculated biological effective dose.

In this retrospective study we aimed to examine if hypofractionated stereotactic radiotherapy impacts local control of brain metastases from radioresistant tumors such as melanoma and renal cancer.

## Methods

### Patients

Between May 2012 and December 2016, we screened all patients who had received a stereotactic radiotherapy for brain metastases from melanoma or renal cancer in our institution (Centre François Baclesse, Caen, France). This study was approved by the French ethic committees: CEREES and CNIL. An information letter was sent to patients still alive at time of data collection. Histology was confirmed by pathologic analysis, with biopsy from primitive site or from metastases. Patients should have had at least a pre and post treatment MRI evaluation. Metastases bigger than 14140mm^3^ (corresponding to a sphere of 3 cm diameter) were excluded. MRI was systematically performed, to make diagnosis of brain metastases and follow-up MRI to assess the response to treatment and intracranial control. Patients who had received upfront WBRT before SRS or HFSRT were excluded. Patients with performans status (PS) superior to 2 were not included. The different metastases from a same patient could be treated with SRS or HFSRT. Patients could have several stereotactic irradiation sessions during the course of their disease.

### Hypofractionated stereotactic radiotherapy and radiosurgery treatments

Brain metastases were treated using a Cyberknife system (Accuray Inc. ®, Sunnyvale®, California). Patients were positioned supine on the 6D robotic couch and immobilized with a commercial stereotactic mask fixation system. Tracking was performed with the Cyberknife 6D skull system®. All the patients benefited from a 1 mm thick slice CT scan (CT, Philips®, BigBore®) and a 1 mm slice gadolinium-enhanced cerebral MRI (Siemens®). GTV was delineated on axial T1-weighted gadolinium-enhanced MRI. CTV was a zero-margin expansion from GTV and PTV was defined as an isocentric 2 mm expansion from GTV. Doses were prescribed to the 80% isodose line to achieve a minimum 95% target coverage of the prescribed dose. All the metastases treated with a fractionation different from 1, 3 or 6 were also excluded from the analysis. The radiotherapist determined fractionation and the delivered dose after consideration of PTV final volume and proximity of critical structures such as optic chiasma, optic nerves, cavernous sinus, or brain stem for example. Radiosurgery was favored if metastases measured less than 1 cm diameter and far from an organ at risk. Consequently metastases size and localization were the two variables used to build the propension score (cf Statistical analysis). Brain metastases were irradiated on alternate days, every other day. When patients presented several metastases to irradiate, then, they were not irradiated the same day, but the day after.

Salvaged treatments were also evaluated when assessing outcomes. If SRS or HFSRT failed, patients could receive WBRT or neurosurgery. In case of distant cerebral relapse, another session of stereotactic radiotherapy or WBRT could be proposed, at the discretion of the radiotherapist.

### Follow-up and outcomes

Patients were clinically examined one month after treatment, and then every two months. At each medical consultation a gadolinium-enhanced MRI was performed. Duration of follow-up was calculated as time from first fraction of HFSRT or SRS to last MRI evaluation. The primary outcome was local progression-free survival (LPFS: Patient alive without in-field local recurrence). For lesions larger than 1 cm, an increase of enhanced tumor volume superior to 20% was considered as a local progression and a decrease superior to 30% as a partial response, according to Recist1.1 criteria [10]. For lesions, smaller than 1 cm, an unequivocal increase or a decrease of the enhanced tumor volume was needed to conclude to progression or response respectively. Finally, total disappearance of the lesion was considered as a complete response. The diagnosis of radionecrosis was made on the following criteria: 1) increased T1 contrast enhancement located in the irradiated area with central hypo intensity and increased peripheral edema; 2) substantial regression or stability (for at least 4 months) of enhancing areas on serial follow-up MRI scans, without additional treatment. MRI perfusion sequences were not systematically performed. They were performed only if there was some doubt between relapse or radionecrosis, which could potentially lead to a change of care. Conformity, homogeneity, and coverage indexes were reported following ICRU 83 definitions [11]. Gradient index 50 reflecting the dose fall-off was also reported [12].

### Statistical analysis

Patient’s characteristics were described by mean and standard deviation or by median and range for continuous variables and by frequencies for categorical variables. Then, comparisons of these variables, through fractionation, were assessed by the use of Fisher’s exact test and Pearson’s chi-square test for categorical data, and by independent samples t-tests or Mann-Whitney tests as appropriate for normally or non-normally distributed data, respectively. LPFS was defined as the time, for a given metastasis, from the first day of irradiation to the appearance of local failure. Cerebral progression-free survival (CPFS) and overall survival (OS) were defined as the time from the first day of first radiation treatment to the appearance of the first distant cerebral failure and the death from any cause, respectively. For LPFS, patients not having any evidence of local failure on MRI were censored at last MRI.

Survival probability was estimated using the Kaplan-Meier method. Univariate and multivariate analyses using Cox models and log-rank tests were performed to evaluate the effects of various variables on LPFS. To reduce or minimize the effects of confounding variables on LPFS due to the non-randomized data, a propensity score matching method was used in a supplementary analysis [13, 14]. The propensity score, defined in this context as the probability of fractionation assignment given measured baseline covariates, was estimated using a logistic regression model, containing the PTV volume, the tumour size and the time from planning IRM to irradiation as influencing covariates, which are three of the most significant variables. Then, matched samples of lesions treated by SRS and lesions treated by HFSRT were formed such that confounding factors are balanced between both groups. Cox model and log-rank test were applied to original and to the matched samples to evaluate the effect of fractionation on LPFS. An outcome-oriented method based on the maximally selected rank statistic has been used to provide the optimal cut point, corresponding to the most significant relation with LPFS for continuous variables (except GTV), through ‘survminer’ R package [15]. Analyses were performed using R version 3.4.0 [16].

## Results

### Patients’ characteristics

From May 2012 to December 2016, 60 patients underwent stereotactic irradiation for brain metastases from melanoma or renal cancers. The primary tumor was melanoma for 62% of the sample (Table 1). A majority of patients had extra-cerebral metastatic disease (75%) at first course of stereotactic irradiation. The overall condition of patients was good. Indeed 85% of patients had a PS lower or equal to 1, and 81% had a DS-GPA greater or equal to 2. A quarter of the patients included in this study had undergone resection, either as upfront treatment or during the disease progression.

**Table 1:** Characteristics of patients at first stereotactic radiotherapy

Characteristics of patients (n=60)	
Age (Median)[range]	66 years [18–88]
Sex	Female *n* = 24 (40%) / Male *n* = 36 (60%)
Histology	Melanoma *n* = 37 (62%) / Renal cancer *n* = 23 (38%)
Neurosurgery	Yes *n* = 15 (25%) / No *n* = 45 (75%)
Performans status	
0	18 (30%)
1	33 (55%)
2	9(15%)
≥ 3	0
DS GPA	
4	6 (10%)
3	17 (28%)
2	26 (43%)
1	10 (17%)
0	1 (2%)
Whole brain radiotherapy	Yes *n* = 16 (27%)/ No *n* = 44 (73%)
Time From diagnosis to first stereotactic radiotherapy	Median: 55 days, range [15–1420] Mean: 109 days, (sd = 200)
Extra-cerebral disease	Yes n = 45 (75%)/ No *n* = 15 (25%)
Immunotherapy during the course of their disease	Yes *n* = 21(35%)/ No *n* = 39 (65%)

### Metastasis and radiation treatment characteristics

The total number of brain metastases treated was 193. The median count of brain metastases in a single patient was 3 (range [1–11]) irradiated with SRS or HFSRT during the whole course of his/her disease. Fifty-two (27%) metastases were treated with SRS and 141 (73%) with HFSRT (Table 2). Three irradiation schedules were used: 1 fraction (median dose per fraction: 20 Gy [range: 18-25Gy]), 3 fractions (10 Gy [9–11 Gy]) or 6 fractions (6 Gy [5–6 Gy]).For HFSRT treated metastases, the most used schedules were 3 × 10 Gy(*n* = 106; 55%) and 6 × 6 Gy (*n* = 35; 18%)); 91% of these metastases were treated with these schedules. For the SRS group, 88% of the metastases were irradiated with one fraction of 20 Gy or 22 Gy. The median prescription isodose for both groups was 80%. GTV and PTV median volumes were statistically different for SRS treated metastases in comparison with HFSRT: 125 and 474,5 mm^3^ vs 699 and 1752 mm^3^, respectively (*p* < 0.001). PTV coverage was statistically different in the SRS group than in HFSRT group (with median values of 0.99 vs 0.97, *p* = 0.034) as well as for the fall-off in dose (with respectively median values of 5.71 vs 4.39, p < 0.001). For the SRS and HFSRT groups, the proportion of infra-tentorial lesions was 16 and 17% respectively. Median time from fusion MRI to the beginning of irradiation were 26 days and 17 days for SRS and HFSRT treated metastases respectively(p < 0.001). About a third of metastasis (30%) were treated with a delay from fusion MRI less than 14 days. Median delay from simulation CT and first irradiation was 17 days.

**Table 2:** Characteristics of treated metastases and radiotherapy

	SRS (n fraction = 1)	HFSRT (n fraction = 3 or 6)	*p* value
n metastases (total = 193)	52 (27%)	141 (73%)	
		3 fractions *n* = 106 / 6 fractions *n* = 35	
Follow up (mean (sd))	mean: 359 days (315)	mean: 230 days (206)	0.001
Treatment schedules	1x18Gy (*n* = 5)	6x5Gy (*n* = 3)	
	1x20Gy (*n* = 34)	6x6Gy (*n* = 32)	
	1x22Gy (*n* = 12)	3x9Gy (*n* = 8)	
	1x25Gy (*n* = 1)	3x10Gy (*n* = 97)	
		3x11Gy (*n* = 1)	
Dose per fraction	Mean: 20.38 (1.34) Median: 20	Mean: 8.94 (1.77) Median: 10	< 0.001
GTV volume	Mean: 173.70 (183.78) Median: 125	Mean: 1820.40 (2622.22) Median: 699	< 0.001
PTV volume	Mean: 565.37 (407.64) Median: 474.5	Mean: 4118.31 (7895.40) Median: 1752	0,001
Homogeneity Index	Mean: 0.22 (0.07) Median: 0.22	Mean: 0.22 (0.08) Median; 0.21	0.057
Conformity Index	Mean: 1.02 (0.28) Median: 1.04	Mean: 1.01 (0.18) Median: 1.04	0.894
Coverage index	Mean: 0.96 (0.06) Median: 0.99	Mean: 0.94 (0.12) Median: 0.97	0.034
Gradient index 50	Median: 5.71	Median: 4.39	< 0.001
Isodose of prescription (median)	80% [58–96]	80% [75–91]	0.334
Histology			
Melanoma (*n* = 137)	38	99	0.833
Renal cancer (*n* = 56)	14	42	
Localization			
Supra tentorial (*n* = 165)	45	120	0.98
Infra tentorial (*n* = 28)	7	21	
Time from planning IRM to Irradiation (median)	26 days [5–82]	17 days [3–69]	< 0.001

### Local progression free survival based on complete dataset

**Fig. 1:**
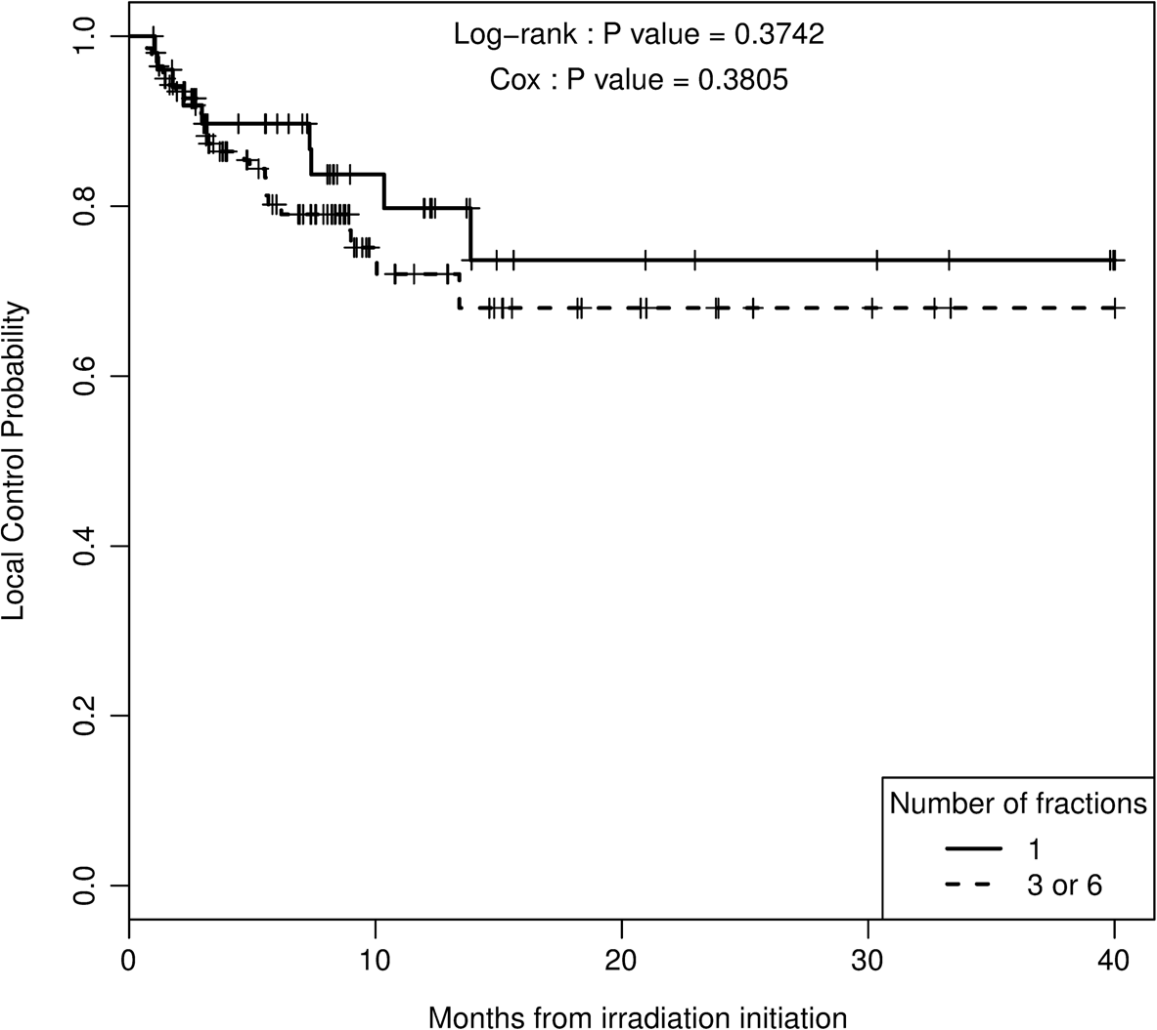
Local control probability function of fractionation

After a median follow-up of 7.4 months, LPFS rates at 6, 12 and 18 months for the whole population were 83, 74 and 70%, respectively. With respect to fractionation, LPFS rates at 6, 12 and 18 months were 89, 79 and 73% for the SRS group and 80, 72 and 68% for the HFSRT group (Fig. 1). The fractionation schedule was not statistically associated with LPFS (HR = 1.39, CI95% [0.65–2.96], *p* = 0.38). The difference in fractionation schemes was also calculated independently for 3 and 6 fractions, and there was not significant difference.

**Fig. 2:**
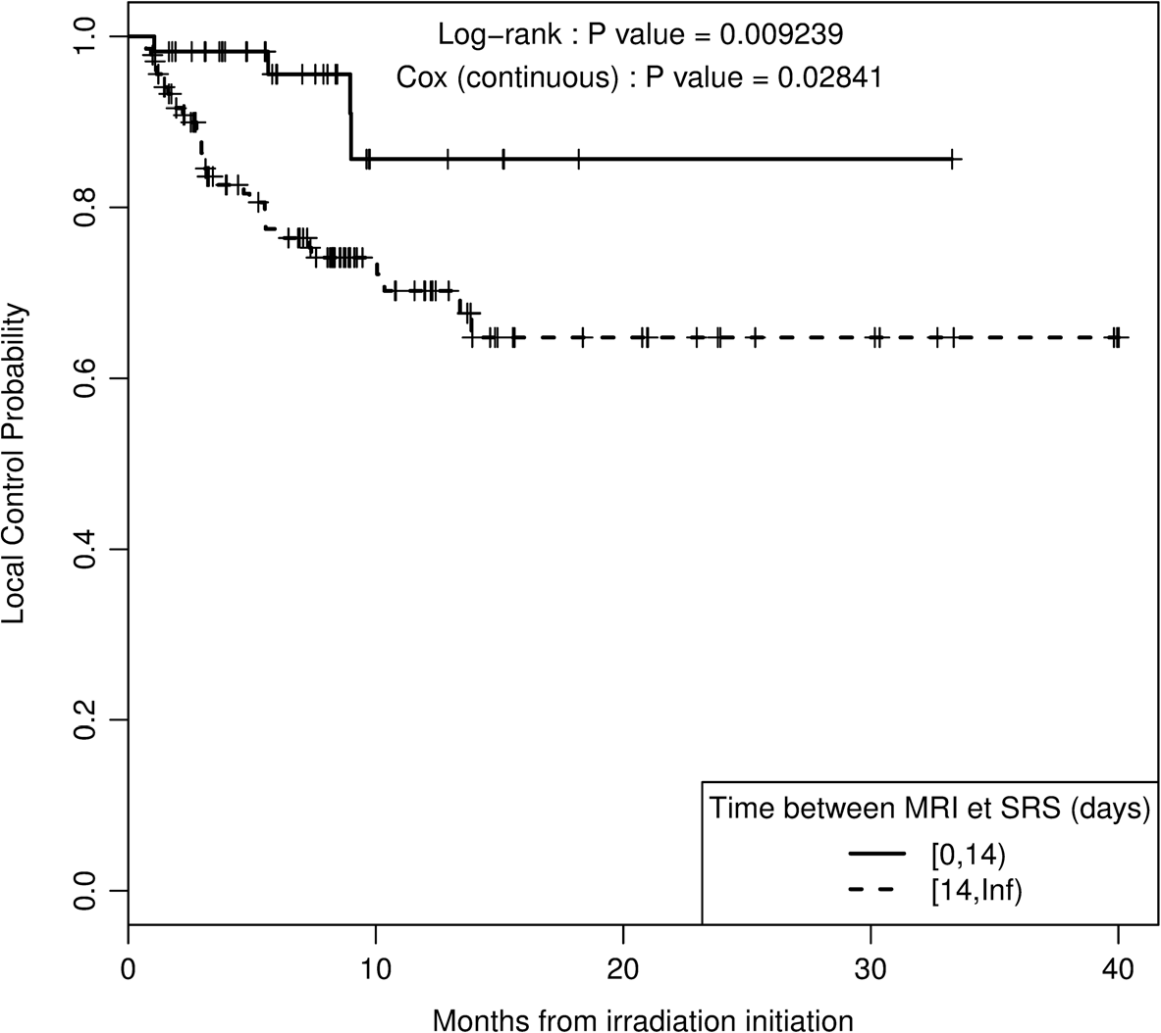
Local control probability function of delay from MRI fusion to first irradiation

Best objective response rates observed were statistically different between both groups (*p* = 0.022), (Table 3). Indeed, the complete response rate was higher in the SRS group than in the HFSRT group (38% vs 21%, *p* = 0.022). However, complete and partial response rates were similar, about 48%, for both groups. Results of univariate and multivariate analyses are displayed in Table 4. Especially, a time from planning MRI to first irradiation session longer than 14 days was associated with a poorer local control rate (Fig. 2). Over this time, LPFS at 12 months was reduced from 86 to 70% (*p* = 0.009). Furthermore, a GTV volume higher than 530 cm^3^, corresponding to a 1 cm diameter sphere, was associated with an increased risk of local failure, from 17 to 36% (*p* = 0.007) at 12 months. Finally, univariate analysis showed that coverage index lower than 0.985 was predictive of local relapse (*p* = 0.019). Each of these three factors was also significantly associated with LPFS in a multivariate model, with a global likelihood ratio test *p*-value < 0.001. None of the other histologic, dosimetric, imaging or clinical factors tested were predictive of local failure (at a 5% level of significance). Particularly, in the melanoma sub group, BRAF mutation did not impact outcome of stereotactic radiotherapy (*p* = 0.41).

**Table 3:** Effect of SRS or HFSRT on local control, toxicity and objective response

Effect of SRS or HFSRT on local control			
Local Control	Hazard Ratio (HR)	IC 95%	p
Unadjusted cohort	1.39	[0.65–2.96]	0.38
Pairs matched propensity score	1.91	[0.66–5.5]	0.22
Effect of SRS or HFSRT on toxicity and objective response			
	SRS	HFSRT	p
Radionecrosis (%)	5 (9.6)	10 (7.1)	0,554
Hematoma (%)	0 (0.0)	4 (2.8)	0,576
Best.Reponse.objective (%)			0.022
Complete	20 (38.5)	30 (21.3)	
Partial response	5 (9.6)	37 (26,2)	
Progression	6 (11.5)	14 (9.9)	
Stable	21 (40.4)	60 (42.6)

**Table 4:** Univariate and Multivariate analysis of covariates influencing local control

	Univariate analysis			Multivariate analysis^a^		
	HR	CI 95%	*p*-value	HR	CI 95%	*p*-value
Time from MRI to first irradiation (ref < 14 days)	3.60	[1.27;10.19]	0.009	3.98	[1.40;11.27]	0.003
GTV (ref < 530)	2.44	[1.24;4.80]	0.007	2.30	[1.16;4.55]	0.014
Coverage Index (ref < 0.985)	0.43	[0.21;0.89]	0.019	0.46	[0.22;0.95]	0.029

^a^global likelihood ratio test p-value< 0.001

### Propensity score matching analysis

In a supplementary analysis, a propensity score was calculated to achieve balanced distribution of baseline characteristics and confounding variables in treatment groups in order to compare LPFS after SRS or HFSRT. Confusing variables was tumor size, PTV volume, and delay from MRI to irradiation. Propensity score matching resulted in 42 matched pairs, whose characteristics are displayed in Additional file 1: Table S1, showing that the treatment groups were well balanced across all covariates. No significant differences in LPFS between groups were observed, meaning that fractionation did not alter local control (HR 1.91 [0.66–5.90], *p* = 0.22). Analysis of prognostic factors showed that GTV volume (> or < 530 mm^3^) was still associated with LPFS in the matched pairs samples (HR 8.43 [3.04–23.32], *p* < 0.001), although GTV was no more significantly different between groups (*p* = 0.17). Delay from MRI to irradiation still appears as a prognostic factor on LPFS, with however a longer optimal cut-off of 24 days (HR 5.8 [1.30–25.8], *p* = 0.009). Note that a continuous Cox model analysis show that the delay from MRI to irradiation considered as a continuous variable is significantly associated with LPFS for both complete and matched pairs samples (*p* = 0.03 and *p* = 0.02 respectively), with a poorer local control for long delays. Coverage index was no more associated with LPFS (*p* = 0.47).

### Overall survival

**Fig. 3:**
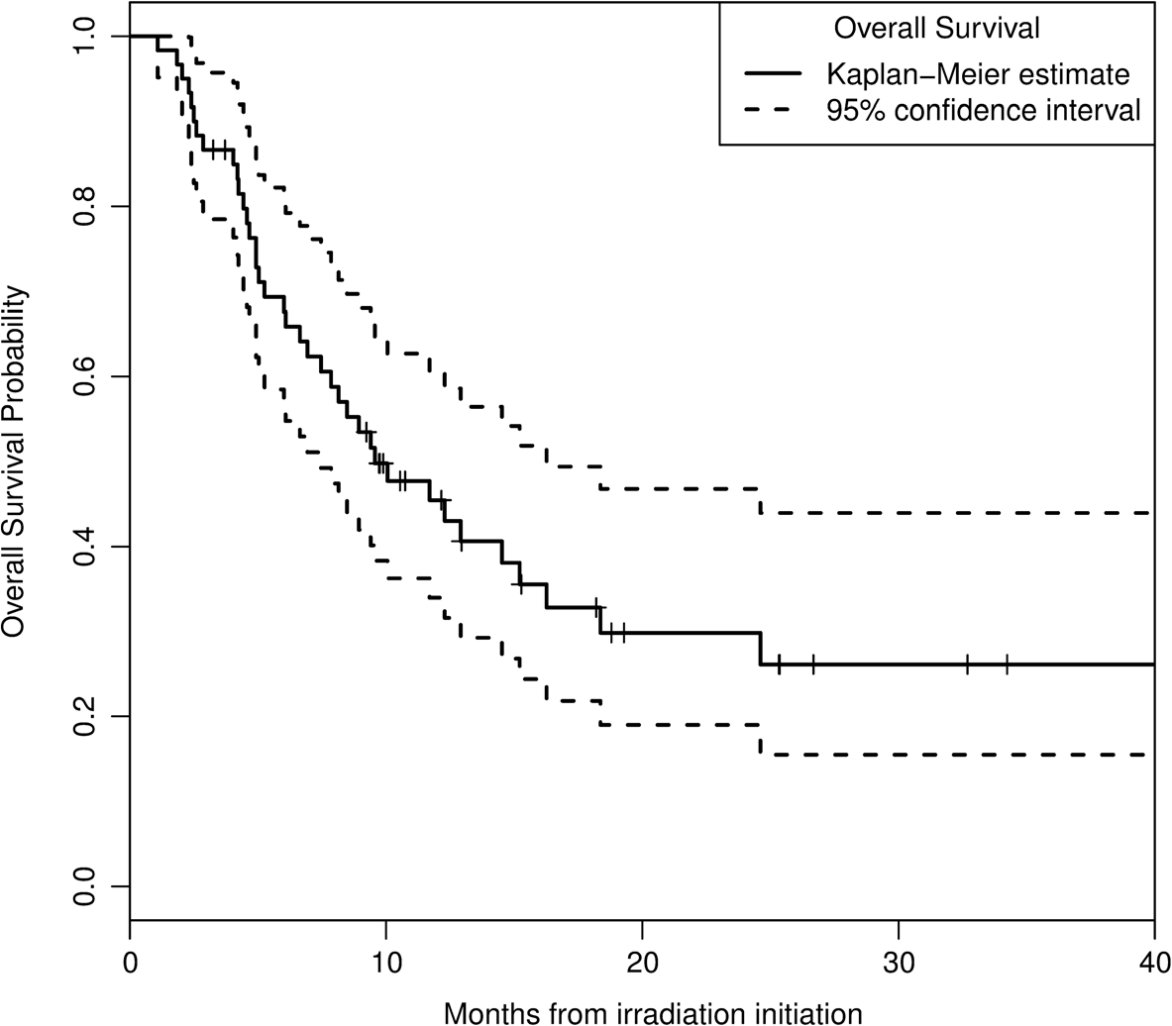
Overall survival

The median OS was 9.6 months and survival rates at 6, 12 and 18 months were 69, 45 and 33%, respectively (Fig. 3). Histology had no impact on OS (*p* = 0.42). DS-GPA lower or equal to 2 was of borderline significance for predicting poor overall survival (*p* = 0.11). As it was supposed to, patients who had received immunotherapy (anti PD1 or Anti-CTLA-4) during the course of their disease lived much longer than those who did not (12 months OS rate: 72% vs 31%, *p* = 0.002).

### Cerebral progression free survival

Six, 12 and 18 months distant brain failure rates were 54, 24 and 17%, respectively (Fig. °4). Upon univariate analysis, patients with more than 3 metastases at first irradiation course had a higher risk of distant cerebral failure (*p* = 0.03). For these patients, the relapse rate was 91% at 12 months. Patients with melanoma trended to have a worse CPFS in comparison with patients with renal cancer (*p* = 0.14). None of the following factors was predictive of distant cerebral relapse: age, sex, DS-GPA, performance status, presence of extra-cerebral disease, or a history of neurosurgery for brain metastases. In case of brain distant relapse, a new stereotactic irradiation session could be proposed.

**Fig. 4:**
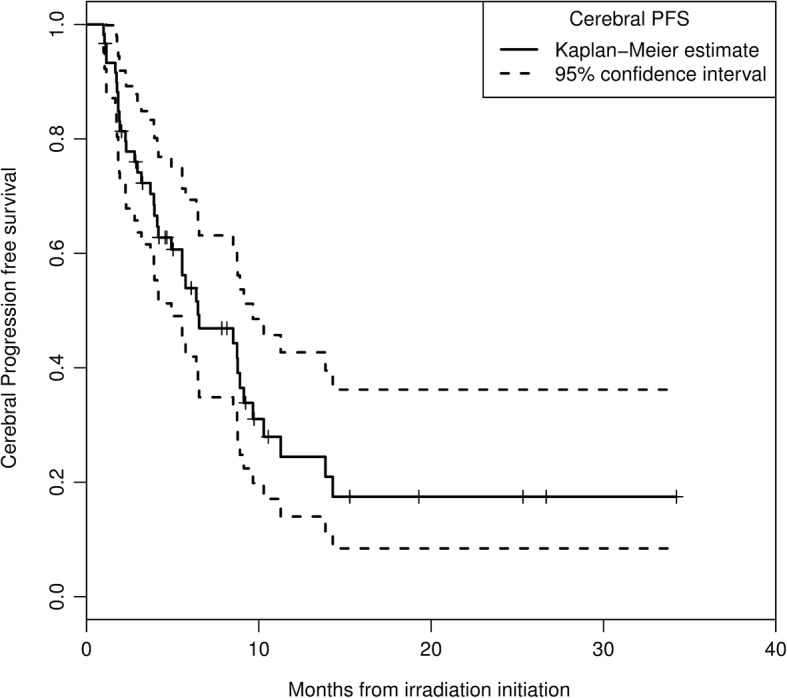
Cerebral Progression Free Survival

Thus 28 and 9% of the patients received two or three courses of stereotactic radiotherapy. The remaining two thirds received only one course of stereotactic radiotherapy (63%).

### Side effects of stereotactic radiotherapy: Radionecrosis and hematoma

Radionecrosis occurred in 7.1% for HFSRT treated metastases to 9.6% to SRS treated metastases, without any difference according to fractionation (*p* = 0.55) (Table 3). Appearance of intra-tumoral post treatment hemorrhage occurred in four metastases (2%): two from melanoma and two from renal cancer. These side effects were diagnosed on follow-up MRI and patients were strictly asymptomatic. They did not need any medical care.

## Discussion

This single center retrospective study has been led in order to explore the impact of stereotactic radiotherapy fractionation on local control of small radioresistant brain metastases. The analysis was conducted on 60 patients and a total of 193 metastases smaller than 3 cm with a median follow-up of 7.4 months between 2013 and 2016. To the best of our knowledge, it is the largest published collection, dealing with the impact of fractionation on local control of only radioresistant brain metastases. Local control at 12 months was 74%, closer to control rates reported in recent retrospective studies, which range from 81 to 91,5% [7, 16–23].

Whereas until now, there was mounting evidence that HFSRT was an effective approach in terms of local control for larger brain metastases or radiosensitive histologies [24], similar results, to our knowledge, have not been mentioned for smaller and radioresistant brain metastases.

On the basis of the linear quadratic (LQ) model [25], considering an α/β ratio of about 2.5, SRS patients received a median BED_2.5_ of 180Gy and HFSRT patients a BED_2.5_ from 122Gy for the 6 × 6 Gy schedule to 150Gy for the 3 × 10 Gy schedule. Unexpectedly, better local control was not observed in the SRS subgroup as HFSRT was equivalent to SRS in terms of local control. These results are quite different and contradictory with Oermann’s [8]. In fact, in a smaller study with a subgroup of 99 radioresistant brain metastases, he described a trend toward improved local control for single-fraction radiosurgery [8]. However, even if dose per fraction was not reported in that study, the median delivered dose was 20Gy. It was much less than in our study where patients in the HFSRT group received a median dose of 30Gy. Higher dose per fraction and total dose in HFSRT group improve the efficacy of fractionated radiotherapy.

In our series, the LQ model was clearly not predictive of local control, and we believe that LQ model is inadequate in these situations. Indeed, LQ model is based on the assumption that radiation-induced cell death in tumor is due to DNA strand breaks. Then LQ model underestimates the role of indirect cell death by devascularization and radiation induced immune enhancement [26, 27]. Radiation-induced apoptosis of endothelial cells, leading to indirect tumor cell death, is predominant for dose per fraction higher to 10 Gy [28], and consequently, here, it could not explain our results. Concerning radiation immune effects, recent pre-clinical studies support a superior anti tumoral immune enhancement effect for high dose fractionated radiotherapy (>8Gy) than for single fraction irradiation [29, 30]. Here, our study dealt with melanoma and renal cancers, which are classified as ‘immunogenic’ tumor, based on several characteristics: incidence of spontaneous tumor regression, high level of tumor T-cell infiltration and responsiveness to immunotherapies such as interleukin 2 (IL-2) interferon alpha (IFN-α) or Anti PD1 [31]. On these arguments, the “unexpected” good local control rate for HFSRT-treated metastases could be due to a better radiation-induced immune enhancement. Nevertheless, this hypothesis is based on pre-clinical studies or case reports studies, and should be studied and discussed in prospective trials [32, 33].

One of the other interesting points raised in our analysis concerned the delay in initiating radiotherapy after fusion MRI. When radiotherapy was delayed from more than 14 days after fusion MRI, local control at 6 and 12 months decreased from 96 and 86% to 77 and 70% respectively (*p* = 0.009). For these patients, delays in workflow could make pretreatment imaging inadequate for SRS or HFSRT planning, and thus, as the tumor continues to grow the reduced margins used in these treatments, become insufficient to cover the whole target. The same cut off of 14 days was reported by Seymour et al. [34]*.* In this study, the 6- and 12-month LPFS rate were 95 and 75% for metastasis with interval of < 14 days from MRI to treatment compared to 56 and 34% for metastases with MRI ≥ 14 days before treatment. In our study, fusion MRI was obtained on the same day as simulation for 31% (*n* = 60) of mestastases. For 27% (*n* = 52) of metastases, MRI was obtained after simulation, and for 42% of metastases (*n* = 81) a new MRI was not acquired at or after simulation. In this case, the diagnostic MRI, with sufficient transverse images was used. This last group participate to extend delay from MRI to treatment and could explain extreme values and why the delay is quite long in our serie. The extreme values corresponded to the first patients treated with the Cyberknife in our institution on January 2013. To conclude, time from MRI to SRS or HFSRT should be the shortest as possible, and delaying the beginning of the treatment should be absolutely avoided, as confirmed in propensity score matching analysis.

OS and CPFS remain disappointing, and stereotactic radiotherapy could not be considered as a single modality treatment. Poor CPFS was expected. In fact, stereotactic radiotherapy offers to patients good local control rate, while avoiding neurocognitive late effects, but with more cerebral distant relapse in comparison with SRS/HFSRT plus WBRT [4].Nevertheless, when cerebral distant relapse occurs, HFSRT/SRS rescue treatment still remains possible and WBRT can be delayed. For example, in our study, 37% of the patients had received a second or a third session of stereotactic irradiation with excellent outcomes. And for only 27% of the patients, a rescue WBRT was needed.

If this strategy offers satisfactory cerebral control, the association with efficient systemic treatments such as anti-PD-1 RAF/MEK inhibitors, or anti-angiogenic is essential, to improve extra cerebral control and overall survival. In our sample, for example, 35% of patients received at least one immunotherapy (nivolumab, pembrolizumab or ipilimumab) and for them, overall survival was significantly extended (12 months OS rate: 72% vs 31%, *p* = 0.002). In contrast, it is worth to note that, here, LPFS and CPFS were not improved with immunotherapy. Thus, immunotherapy, in our sample, probably, increased OS by improving extra cerebral control rate.

The main limitation of our study is its retrospective nature. It suffers from the shortcomings of all retrospective studies. The two compared groups are not strictly similar; the major difference being the size of the target volumes. For example, in SRS group, GTV volumes were quite smaller than HFSRT group. Assignment to single fraction irradiation or HFSRT was not random and based on GTV volume and localization. It could lead to selection biases in terms of treatment choice. However, to limit this selection bias, we performed a propensity score matching analysis, which makes the comparison more robust (the two experimental groups were comparable), and lead to similar conclusions. Furthermore, in our study, the radionecrosis rate was quite low, about 9%, whatever the fractionation. For HFSRT treated metastases, this rate was similar to others studies [24, 35, 36]. On the opposite, contrary to our results, for SRS treated metastases, several authors reported radionecrosis rates much higher than our study with rate superior to 20% [24, 37]. This difference could be explained by different ways. First, given the poor overall survival of our population, follow-up (7,4 months) was too short to see the emergence of fewer radionecrosis. In Minniti’s [24] and Kohutek’s [37] studies, follow-up were much longer, 29 months and 17.2 months respectively, but with a high proportion of non-small cell lung cancer brain metastases. Most of the time, radionecrosis occurs 10 to 12 months after stereotactic radiotherapy [37]. Secondly, in our institution, multi-parametric MRI or F-DOPA PET-scan were not systematically performed during the post stereotactic radiotherapy brain metastases follow-up period. Thus, our diagnosis criteria were probably less sensitive than in other studies to detect radionecrosis.

## Conclusion

As a conclusion, this is the largest retrospective study evaluating the impact of stereotactic radiotherapy fractionation on radioresistant brain metastases. Fractionation does not seem to decrease LPFS. Even for radioresistant smaller brain metastases (< 3 cm), HFSRT, with 3 or 6 fractions, leads to an excellent local control rate of 72% at 1 year with only 7.1% rate of radionecrosis. HFSRT is a safe and efficient alternative treatment to SRS. In case of proximity of highly functional zones, such as brainstem or cavernous sinus, the radiotherapist can safely propose a 3 or 6 fractions schedule, subject to dosimetric constraints respect, without compromising local control, even if the metastasis is small.

These data have to be confirmed by a prospective randomized trial. Whatever the irradiation fractionation, the radiotherapist should make the necessary efforts to begin the treatment less than 14 days after MRI fusion, in order to ensure optimal local rate.

## Additional file

Additional file 1: **Table S1.** Characteristics of treated metastases and radiotherapy for the pairs matched groups. (DOCX 16 kb)

## Abbreviations

CPFS: Cerebral progression Free survival
GTV: Gross tumor volume
HFSRT: Hypofractionated stereotactic radiotherapy
LPFS: Local progression free survival
MRI: Magnetic resonance imaging
OS: Overall Survival
PS: Performance status
PTV: Planification target volume
SRS: Radiosurgery

## References

[1] Flanigan RC, Campbell SC, Clark JI, Picken MM (2003). Metastatic renal cell carcinoma. Curr Treat Options in Oncol.

[2] Tas F. Metastatic Behavior in Melanoma: Timing, Pattern, Survival, and Influencing Factors. J Oncol. 2012;2012 Available from: https://www.ncbi.nlm.nih.gov/pmc/articles/PMC3391929/10.1155/2012/647684 PMC3391929 22792102

[3] Yamamoto M, Serizawa T, Shuto T, Akabane A, Higuchi Y, Kawagishi J (2014). Stereotactic radiosurgery for patients with multiple brain metastases (JLGK0901): a multi-institutional prospective observational study. Lancet Oncol.

[4] Brown PD, Jaeckle K, Ballman KV, Farace E, Cerhan JH, Anderson SK (2016). Effect of radiosurgery alone vs radiosurgery with whole brain radiation therapy on cognitive function in patients with 1 to 3 brain metastases: a randomized clinical trial. JAMA.

[5] Fowler JF (1989). The linear-quadratic formula and progress in fractionated radiotherapy. Br J Radiol.

[6] Overgaard J (1986). The role of radiotherapy in recurrent and metastatic malignant melanoma: a clinical radiobiological study. Int J Radiat Oncol Biol Phys.

[7] Ning S, Trisler K, Wessels BW, Knox SJ (1997). Radiobiologic studies of radioimmunotherapy and external beam radiotherapy in vitro and in vivo in human renal cell carcinoma xenografts. Cancer.

[8] Oermann EK, Kress M-AS, Todd JV, Collins BT, Hoffman R, Chaudhry H (2013). The impact of radiosurgery fractionation and tumor radiobiology on the local control of brain metastases. J Neurosurg.

[9] Kondziolka D (2013). The biological advantage of single-session radiosurgery. J Neurosurg.

[10] Eisenhauer EA, Therasse P, Bogaerts J, Schwartz LH, Sargent D, Ford R (2009). New response evaluation criteria in solid tumours: revised RECIST guideline (version 1.1). Eur J Cancer Oxf Engl 1990.

[11] Grégoire V, Mackie TR (2011). State of the art on dose prescription, reporting and recording in intensity-modulated radiation therapy (ICRU report no. 83). Cancer Radiother J Soc Francaise Radiother Oncol..

[12] Paddick I, Lippitz B (2006). A simple dose gradient measurement tool to complement the conformity index. J Neurosurg.

[13] Rosenbaum PR, Rubin DB (1983). The central role of the propensity score in observational studies for causal effects. Biometrika.

[14] Rosenbaum PR (1987). Model-based direct adjustment. J Am Stat Assoc.

[15] Kassambara A, Kosinski M, Biecek P, Fabian S (2017). survminer: Drawing Survival Curves using “ggplot2” [Internet].

[16] R Core Team. R: A language and environment for statistical computing. Vienna: R Foundation for Statistical Computing; 2013. http://www.R-project.org/.

[17] Lwu S, Goetz P, Monsalves E, Aryaee M, Ebinu J, Laperriere N (2013). Stereotactic radiosurgery for the treatment of melanoma and renal cell carcinoma brain metastases. Oncol Rep.

[18] Ahmed KA, Sarangkasiri S, Chinnaiyan P, Sahebjam S, Yu H-HM, Etame AB (2016). Outcomes following Hypofractionated stereotactic radiotherapy in the Management of Brain Metastases. Am J Clin Oncol.

[19] Rades D, Huttenlocher S, Gebauer N, Hornung D, Trang NT, Khoa MT (2015). Impact of stereotactic radiosurgery dose on control of cerebral metastases from renal cell carcinoma. Anticancer Res.

[20] Yaeh A, Nanda T, Jani A, Rozenblat T, Qureshi Y, Saad S (2015). Control of brain metastases from radioresistant tumors treated by stereotactic radiosurgery. J Neuro-Oncol.

[21] Frakes JM, Figura NB, Ahmed KA, Juan T-H, Patel N, Latifi K (2015). Potential role for LINAC-based stereotactic radiosurgery for the treatment of 5 or more radioresistant melanoma brain metastases. J Neurosurg.

[22] Tuleasca C, Negretti L, Faouzi M, Magaddino V, Gevaert T, von Elm E, et al. Radiosurgery in the management of brain metastasis: a retrospective single-center study comparing gamma knife and LINAC treatment. J Neurosurg. 2018;128(2):352–61. 10.3171/2016.10.JNS161480 28338441

[23] Kotecha R, Miller JA, Venur VA, Mohammadi AM, Chao ST, Suh JH, et al. Melanoma brain metastasis: the impact of stereotactic radiosurgery, BRAF mutational status, and targeted and/or immune-based therapies on treatment outcome. J Neurosurg. 2018;129:50–9. 10.3171/2017.1.JNS162797 28799876

[24] Minniti G, Scaringi C, Paolini S, Lanzetta G, Romano A, Cicone F (2016). Single-fraction versus multifraction (3 × 9 Gy) stereotactic radiosurgery for large (>2 cm) brain metastases: a comparative analysis of local control and risk of radiation-induced brain necrosis. Int J Radiat Oncol Biol Phys.

[25] Brown JM, Carlson DJ, Brenner DJ (2014). The tumor radiobiology of SRS and SBRT: are more than the 5 Rs involved?. Int J Radiat Oncol Biol Phys.

[26] Sperduto PW, Song CW, Kirkpatrick JP, Glatstein E (2015). A hypothesis: indirect cell death in the radiosurgery era. Int J Radiat Oncol Biol Phys.

[27] Kirkpatrick JP, Meyer JJ, Marks LB (2008). The linear-quadratic model is inappropriate to model high dose per fraction effects in radiosurgery. Semin Radiat Oncol.

[28] Garcia-Barros M, Paris F, Cordon-Cardo C, Lyden D, Rafii S, Haimovitz-Friedman A (2003). Tumor response to radiotherapy regulated by endothelial cell apoptosis. Science.

[29] Dewan MZ, Galloway AE, Kawashima N, Dewyngaert JK, Babb JS, Formenti SC (2009). Fractionated but not single-dose radiotherapy induces an immune-mediated abscopal effect when combined with anti-CTLA-4 antibody. Clin Cancer Res Off J Am Assoc Cancer Res.

[30] Demaria S, Golden EB, Formenti SC (2015). Role of local radiation therapy in Cancer immunotherapy. JAMA Oncol.

[31] Itsumi M, Tatsugami K (2010). Immunotherapy for renal cell carcinoma. Clin Dev Immunol.

[32] Gandhi SJ, Minn AJ, Vonderheide RH, Wherry EJ, Hahn SM, Maity A (2015). Awakening the immune system with radiation: optimal dose and fractionation. Cancer Lett.

[33] Barker CA, Postow MA (2014). Combinations of radiation therapy and immunotherapy for melanoma: a review of clinical outcomes. Int J Radiat Oncol Biol Phys.

[34] Seymour ZA, Fogh SE, Westcott SK, Braunstein S, Larson DA, Barani IJ (2015). Interval from imaging to treatment delivery in the radiation surgery age: how long is too long?. Int J Radiat Oncol Biol Phys.

[35] Doré M, Martin S, Delpon G, Clément K, Campion L, Thillays F (2017). Stereotactic radiotherapy following surgery for brain metastasis: predictive factors for local control and radionecrosis. Cancer Radiother J Soc Francaise Radiother Oncol.

[36] Minniti G, D’Angelillo RM, Scaringi C, Trodella LE, Clarke E, Matteucci P (2014). Fractionated stereotactic radiosurgery for patients with brain metastases. J Neuro-Oncol.

[37] Kohutek ZA, Yamada Y, Chan TA, Brennan CW, Tabar V, Gutin PH (2015). Long-term risk of radionecrosis and imaging changes after stereotactic radiosurgery for brain metastases. J Neuro-Oncol.

