# Supplementary material for: Radiosurgery or hypofractionated stereotactic radiotherapy for brain metastases from radioresistant primaries (melanoma and renal cancer)
Source: Radiat Oncol. 2018 Jul 28;13:138. doi: 10.1186/s13014-018-1083-1 (PMC6064124; doi:10.1186/s13014-018-1083-1)
Supplement: Supplementary file 1 — Table S1. Characteristics of treated metastases and radiotherapy for the pairs matched groups. (DOCX 16 kb) [file 13014_2018_1083_MOESM1_ESM.docx]

| **CHARACTERISTICS OF TREATED METASTASES AND RADIOTHERAPY** | | | |
| --- | --- | --- | --- |
|  | **SRS (n fraction= 1)** | **HFSRT (n fraction = 3 or 6)** | **p value** |
| **n metastases (total = 84)** | 42 | 42 |  |
|  |  | 3 fractions n= 39 / 6 fractions n= 3 |  |
| **Follow up (mean (sd))** | mean : 371 days (343)  Median : 252 | mean : 199 days (164)  Median : 193 | 0.021 |
| **Treatment schedules** | 1x18Gy (n=5) | 3x9Gy (n=4) |  |
|  | 1x20Gy (n=27) | 3x10Gy (n=35) |  |
|  | 1x22Gy (n=10) | 6x6Gy (n=3) |  |
|  |  |  |  |
| **GTV volume** | Mean : 195.07 (196.96)  Median : 139.5 | Mean : 264.78 (275.88)  Median : 184.5 | 0.17 |
| **PTV volume** | Mean : 615.95 (430.91)  Median : 519.5 | Mean : 808.55 (560.28)  Median : 704 | 0.057 |
| **Homogeneity Index** | Mean : 0.23 (0.07)  Median : 0.22 | Mean : 0.24 (0.1)  Median ; 0.22 | 0.58 |
| **Conformity Index** | Mean : 1.08 (0.24)  Median : 1.04 | Mean : 0.98 (0.2)  Median : 1.03 | 0.11 |
| **Coverage index** | Mean : 0.97 (0.06)  Median : 0.99 | Mean : 0.93 (0.16)  Median : 0.98 | 0.49 |
| **Isodose of prescription (median)** | 80% [58-95] | 80% [75-86] | 0. 41 |
| **Histology** |  |  |  |
| Melanoma ( n=61) | 29 | 32 | 0.46 |
| Renal cancer (n= 23) | 13 | 10 |  |
| **Time from planning IRM to Irradiation (median)** | 25 days [5-68] | 24 days [5-69] | 0.89 |

Table S1
